# Identifying critical inhalation technique errors in Dry Powder Inhaler use in patients with COPD based on the association with health status and exacerbations: findings from the multi-country cross-sectional observational PIFotal study

**DOI:** 10.1186/s12890-023-02566-6

**Published:** 2023-08-17

**Authors:** Janwillem Kocks, Sinthia Bosnic-Anticevich, Joyce van Cooten, Jaime Correia de Sousa, Biljana Cvetkovski, Richard Dekhuijzen, Lars Dijk, Marina Garcia Pardo, Asparuh Gardev, Radosław Gawlik, Iris van der Ham, Ymke Janse, Federico Lavorini, Tiago Maricoto, Jiska Meijer, Boyd Metz, David Price, Miguel Roman Rodriguez, Kirsten Schuttel, Nilouq Stoker, Ioanna Tsiligianni, Omar Usmani, Jaco Voorham, Marika T. Leving

**Affiliations:** 1 General Practitioners Research Institute, Professor Enno Dirk Wiersmastraat 5, 9713 GH Groningen, The Netherlands; 2 University of Groningen, University Medical Center Groningen, GRIAC Research Institute, Groningen, The Netherlands; 3 Observational and Pragmatic Research Institute, Singapore, Singapore; 4 Department of Pulmonology, University of Groningen, University Medical Center Groningen, Groningen, The Netherlands; 5 Woolcock Institute of Medical Research, University of Sydney, Sydney, Australia; 6 Sydney Local Health District, Sydney, Australia; 7 Life and Health Sciences Research Institute (ICVS), PT Government Associate Laboratory, School of Medicine, University of Minho, Braga, Portugal; 8 Radboud University Medical Center, Nijmegen, Netherlands; 9 Primary Care Respiratory Research Unit, Instituto De Investigación Sanitaria De Baleares (IdISBa), Palma, Spain; 10 Boehringer Ingelheim International GmbH, Ingelheim Am Rhein, Germany; 11 Department of Internal Medicine, Allergology, Clinical Immunology, Medical University of Silesia, Katowice, Poland; 12 Department of Clinical and Experimental Medicine, Careggi University Hospital, Florence, Italy; 13 Faculty of Health Sciences, University of Beira Interior, Covilha, Portugal; 14 Centre of Academic Primary Care, Division of Applied Health Sciences, University of Aberdeen, Aberdeen, UK; 15 Department of Social Medicine, Health Planning Unit, Faculty of Medicine, University of Crete, Rethymno, Greece; 16 Airway Disease, National Heart and Lung Institute (NHLI), Imperial College London and Royal Brompton Hospital, London, UK; 17 Data to Insights Research Solutions, Lisbon, Portugal

**Keywords:** Chronic Obstructive Pulmonary Disease, Inhaler technique, Inhaler errors, COPD health status, Exacerbation

## Abstract

**Background:**

Correct inhaler use depends on a complex interplay of factors, including device preparation and generating sufficient inspiratory flow. It is currently unknown which inhalation technique errors can be considered critical in Chronic Obstructive Pulmonary Disease (COPD) patients on Dry Powder Inhaler (DPI) maintenance therapy.

**Objective:**

To investigate the association between inhalation technique errors and health status or exacerbations in patients with COPD. Additionally, the association between the number of errors and COPD outcomes was determined.

**Methods:**

The PIFotal study is a cross-sectional multi-country observational study in a primary care setting, including 1434 COPD patients aged ≥ 40 years (50.1% female; mean age 69.2 yrs) using a DPI for their maintenance therapy. Inhalation technique was video recorded and scored by two independent researchers using inhaler-specific checklists. Health status was assessed with two questionnaires; the Clinical COPD Questionnaire (CCQ) and the COPD Assessment Test (CAT). The number of moderate and severe exacerbations in the past 12 months was recorded. Critical errors were identified based on their association with health status or exacerbations through multi-level prediction models adjusted for identified confounding.

**Results:**

Errors in inhalation technique steps ‘Breathe in’, ‘Hold breath’, and ‘Breathe out calmly after inhalation’ were significantly associated with poorer CCQ and CAT outcomes and thus deemed critical. None of the errors were significantly associated with moderate exacerbations. Patients with errors ‘Preparation’, ‘Hold inhaler in correct position during inhalation’, and ‘Breathe in’ had significantly more severe exacerbations, and therefore these errors were also deemed critical. 81.3% of patients with COPD made at least one critical error. Specific combinations of errors were associated with worse outcomes. The more inhalation technique errors identified, the poorer the health status and the higher the exacerbation rate.

**Conclusion:**

In this study, we identified multiple critical inhalation technique errors in COPD patients using DPIs each associated with poorer outcomes. Explorative analysis revealed that specific combinations of errors may be of clinical relevance, especially those related to the inhalation manoeuvre. COPD outcomes worsened with increasing error count. These results warrant further prospective longitudinal studies to establish the effect of correcting these errors on COPD control.

**Trial registration:**

https://clinicaltrials.gov/ct2/show/NCT04532853 (31/08/2020)

**Supplementary Information:**

The online version contains supplementary material available at 10.1186/s12890-023-02566-6.

## Introduction

COPD is a progressive pulmonary disease characterized by persistent respiratory symptoms and airflow limitation [1]. It is a leading cause of morbidity and mortality that impacts the lives of approximately 384 million patients worldwide [1, 2]. Pharmacological maintenance therapy is primarily prescribed to reduce symptoms and to prevent exacerbations. Long-acting bronchodilators form the primary pharmacotherapeutic treatment in COPD maintenance therapy and are most frequently administrated using Dry Powder Inhalers (DPIs) [1, 3]. Incorrect use of inhalers, in particular DPIs, is a substantial problem among COPD patients [4, 5]. Since DPIs are breath-actuated, patients need sufficient inspiratory flow to achieve optimal drug delivery into the airways. Moreover, correct preparation and handling of the device before inhalation is required and key to treatment success [3, 6, 7].

The PIFotal study determined the association of Peak Inspiratory Flow (PIF), inhalation technique and adherence with COPD outcomes in patients with COPD using DPIs for their maintenance therapy. A suboptimal peak inspiratory flow (PIF) was common (in 29% of the study population) and found to be significantly associated with poor health status and therefore deemed a critical inhaler error in COPD [8]. A considerable proportion (16%) of participants were able to achieve an optimal PIF when instructed to use maximum effort, but failed to do so during a typical inhalation manoeuvre [8]. In light of the importance of PIF for inhaler selection, and the room for improvement in cases of poor technique, objective measurements of PIF have been proposed to help healthcare professionals (HCPs) selecting the most appropriate DPI for their patients [9, 10]. This approach was supported by a post hoc analysis of the PIFotal study, revealing that observations alone were inadequate to identify a suboptimal PIF; when PIF was observed to be sufficient by trained observers, 40% of the patients had a suboptimal PIF when objectively measured using an In-Check DIAL G16 (Clement Clarke, UK) [11].

PIF is a cornerstone of matching an appropriate DPI to the patient, however, other factors of inhalation technique should also be taken into account [9]. Inhalation errors may lead to insufficient medication dose delivery in the airways and eventually to insufficient targeting of airway inflammation and symptoms [12]. Ultimately, this means that even if the patient is adherent to the prescribed medication regimen, a clinical response may not be sufficiently achieved. Where routine measurement of PIF might not be practiced, although this can be fast and inexpensive, observation of inhaler technique can guide the process of matching a patient with an appropriate inhaler and optimise device use. Therefore, inhaler technique needs to be assessed regularly [1].

The CritiKal study was a landmark study exploring the relationship between observed inhalation technique errors and asthma outcomes, including asthma symptom control and exacerbation rate. The study determined that in asthma patients, using a Turbuhaler or Diskus DPI, ‘insufficient inspiratory effort’ was a common error associated with an increased likelihood of having uncontrolled asthma symptoms and increased exacerbation rate and therefore labelled as ‘critical’ error [13].

A previous study in COPD patients deemed errors ‘critical’ if they could have affected dose delivery to the lungs (i.e., device-independent errors such as ‘insufficient inspiratory effort for the DPI’, ‘exhalation into device before inhalation’, or device-specific errors such as ‘incorrect dose preparation prior to inhalation’) [5]. The definition was determined a-priori based on clinical expertise. Patients who made at least one ‘critical’ inhalation error had a higher rate of severe exacerbations compared with patients who did not make these ‘critical’ errors [5].

The process to identify ‘critical’ errors has shown to be challenging, as literature reviews have reported heterogeneous descriptions of critical inhalation errors [4, 6, 14, 15]. Most studies in COPD patients used pre-defined, non-evidence based, critical error checklists. Definitions were pragmatically based on the quantity and potential impact of errors on drug delivery and clinical outcomes, rather than empirically assessed based on their association with poor health outcomes [5, 7, 15, 16] Previous studies have reported that inhalation errors can affect COPD outcomes, but more empirical evidence is needed. Moreover, it is important to identify which specific inhalation technique errors are associated with poorer COPD outcomes. Historically, to make the clinical interpretation of errors (generic and device-specific) simpler for HCPs, it is important that individual inhalation errors are grouped into distinct steps and considered for all available DPIs in a real-world setting [4].

In addition to the assessment of specific inhalation errors, combinations of errors (i.e., error patterns) and the total number of errors and their association with health status and exacerbations should be studied as they might be of clinical importance in COPD. Inhalation technique errors may be interrelated and collectively attribute to poorer COPD outcomes. For example, patients who struggle to exhale fully before the inhalation manoeuvre might also fail to reach sufficient inspiratory flow for their device, diminishing drug dispersion and fine particle generation from the DPI [17, 18]. Yet, the interplay between inhalation technique errors remains largely unexamined.

The primary objective of this post hoc analysis is to assess which individual inhalation errors are associated with poor health status or more frequent moderate or severe exacerbations. Additionally, the possibility of error patterns being of clinical significance will be investigated by the association of various error combinations with health outcomes. Furthermore, the association between the absolute number of inhalation technique errors and health outcomes will be investigated.

## Methods

### Study design

This research is part of the cross-sectional observational multi-country PIFotal study [8, 19]. Patients were recruited and included in the study between October 2020 and May 2021. PIFotal was registered in a public database prior to execution (clinicaltrials.gov; NCT04532853, 31/08/2020). Local medical ethics committees reviewed and approved the study protocol, and all participants provided written informed consent. A flow chart of study procedures is provided elsewhere[8].

### Study population

To ensure a real-world setting as much as possible, inclusion/exclusion criteria were limited in the PIFotal study [8, 19] Patients were included if they: had a clinical diagnosis of COPD; were aged 40 years or older; were treated with a DPI as maintenance therapy for their COPD in the previous 3 months or longer.

Patients were excluded from the study if they: were unable to give informed consent because of a neurodegenerative decline or illness; they were participating in other trials with COPD medication; they had an exacerbation in the 6 weeks prior to participation; or if they suffered from a life-threatening disease with a life expectancy of < 6 months.

### Inhalation technique evaluation [8]

The inhalation technique was observed and documented by video recording which was rated offline by two independent observers (in batches of 20 videos). Checklists including inhaler-dependent and inhaler-independent errors were used, based on recommendations of the Netherlands Lung Alliance (LAN) (www.inhalatorgebruik.nl) or, if unavailable for specific devices, the Aerosol Drug Management Improvement Team (www.inhalers4u.org). The Inhalation technique was evaluated by grouping errors in steps together in 12 categories (Table 1). Differences between the two independent observers were resolved by discussion. In case non-consensus was reached, a third independent expert arbitrated. The inhalation technique errors were dichotomous variables (‘Yes’ / ‘No’ error observed). Inhalation steps marked as not applicable for the device were considered to be ‘no’ error.

**Table 1:** Grouping of errors into 12 distinct error categories

Inhalation error, defined as incorrect performance of:		Device dependent	Device independent
Er1	Preparation	**X**	
Er2	Remove protective cap	**X**	
Er3	Sit up/stand straight & tilt head		**X**
Er4	Hold inhaler in correct position during preparation	**X**	
Er5	Hold inhaler in correct position during inhalation	**X**	
Er6	Breathe out completely before inhalation		**X**
Er7	Teeth and lips sealed around mouthpiece	**X**	
Er8	Empty mouth before inhalation	**X**	
Er9	Breathe in^a^	**X**	
Er10	Hold breath (for at least 6 s)		**X**
Er11	Breathe out calmly after inhalation		**X**
Er12	Rinse mouth		**X**

^a^For an overview of the device-specific checklists see Table S1

### Outcome measures

The outcome measures for this study were COPD-related health status and the number of moderate and severe exacerbations. COPD-related health status was measured with the 10-item self-administered Clinical COPD Questionnaire (CCQ) [20], consisting of three domains: symptoms, functional status, and mental health. The CCQ-score is the mean score of 10 item-scores, where each item is scored on a 7-point Likert scale (0–6) indicating the severity of symptoms. A higher score indicates worse health status. In addition, the COPD Assessment Test (CAT) was administered. The CAT consists of 8 items with 5-point Likert scales to rate symptoms (e.g. frequency of coughing), disability, quality of sleep, and energy [21]. Similar to the CCQ, a higher CAT score indicates worse health status. Exacerbations in the previous 12 months were self-reported by patients or retrieved from medical records in case of no recollection. Exacerbations were categorised as either moderate (i.e. treated with oral corticosteroids and/or antibiotics without a hospital admission) or severe exacerbations (i.e. requiring a hospital admission).

### Patient characteristics

Patient characteristics (including demographic variables, medication regimen, health status, and the number of exacerbations in the past 12 months) and inhalation technique error frequency were described for the total study population.

### Statistical analysis

#### Association between individual inhalation errors with health status and exacerbations

The primary objective was to analyse associations between individual inhalation errors, as described in Table 1, and the following outcomes: CCQ, CAT, or the number of moderate or severe exacerbations in the past 12 months. Multilevel models were used, allowing a random effect at the general practitioners’ level, to take into account clustering of patients. For continuous outcomes (CCQ and CAT) linear multilevel models were used, estimating the average difference (β) in the absolute score (and 95% confidence intervals [CI]) between patients who made a specific inhalation technique error and those who did not make that error. For the number of moderate or severe exacerbations, multilevel negative binomial regressions models were used, reporting rate ratios (RR) (and 95% CI) comparing the number of exacerbations experienced in the past 12 months by patients who made a specific inhalation technique error with the number experienced by those who did not make this error.

Prediction models were built to assess these associations, with a hierarchical forward selection with a *p*-value threshold of 0.10. The models were built using imputed data (15 imputations) for missing inhalation errors and candidate confounders. After an inhalation error was added to the model, all confounder candidates (Table S2) not yet in the model were evaluated for the maximal change in the coefficient of all inhalation errors in the model. In case this was > 10% for at least one of the errors, the candidate confounder was kept in the model. Subsequently, all inhalation errors' significances were recalculated, and iteratively, those with a *p*-value > 0.20 were removed from the model. An overview of the confounders included in the models can be found in Table S3.

An inhalation technique error was deemed ‘critical’ if a statistically significant (*p*-value < 0.05) association was found between the individual error and one of the outcomes.

#### Assocation between error patterns with health status and exacerbations

The secondary objective was to assess, in an explorative manner, if combinations of individual errors (referred to as ‘patterns’) were associated with the health outcomes (CCQ and CAT) or the number of (moderate or severe) exacerbations. In order to increase statistical power, firstly, prediction models were built considering patterns of inhalation errors after removing the errors that were nonsignificant (*p* > 0.2) in the models with the individual inhalation errors and the four health outcomes (as described above). Subsequently, prediction models were built considering patterns of common inhalation errors with the highest effect sizes.

To increase model robustness, the reported models looking at the association between error patterns and the four outcomes considered error patterns occurring in at least 20 patients. All patterns below that frequency were categorized as ‘others’, to ensure that the total population remained intact.

The patterns were considered as a categorical predictor of the outcomes of interest. The reference group consisted of patients that demonstrated none of the assessed error combinations.

#### Number of inhalation errors

The associations of the absolute number of errors with the outcomes, CCQ, CAT, or the number of moderate or severe exacerbations in the past 12 months were assessed. Only the inhalation errors that were found relevant in the pattern combinations were included in these analyses, and this count was used as a categorical predictor.

#### Sensitivity analyses

Sensitivity analyses were performed to assess if the models using the error patterns were confounded by the decision to consider only patterns with a minimum frequency of 20 patients, potentially discarding relevant patterns. Therefore, the analysis was repeated with minimum frequencies of 15 and 10 patients.

In addition, sensitivity analyses were performed to look into subgroup effects of different device resistances (low/medium–low, medium, medium–high/high resistance).

A sample size calculation was performed before study execution for the main study objectives, and not specifically for the analysis concerning critical inhalation technique errors [19]. All statistical analyses were performed using Stata version 15/MP.

## Results

### Study population

A total of 1,434 patients with COPD from 5 European countries (Greece, the Netherlands, Poland, Portugal, Spain) and Australia were included (for an overview please see Table S2). The patients were on average 69 years old, with approaching equal numbers of female (50.1%) and male (49.9%) participants. Approximately 30% were current smokers. Over 68% of the patients had a BMI ≥ 25. GOLD classification of severity of airflow limitation in COPD was available for 801 patients and classified as GOLD stage I in 23.6%, II in 54.9%, III in 17.4%, and IV in 4.1% (Table 2). Of the total study sample, the average CCQ score was 1.7 (SD ±1.1), the average CAT score was 13.6 (SD ± 7.8). In the previous 12 months, 680 moderate and 77 severe exacerbations were reported in 331 participants.

**Table 2:** Overview of participant characteristics

**Variable**		**Total (n = 1434)**
*Age (years)*	Mean (SD)	69.2 (9.3)
*Sex*	Male n (%)	716 (49.9)
	Female n (%)	718 (50.1)
*Body Mass Index (kg/m*^*2*^*)*	< 18.5, n (%)	22 (1.5)
	18.5- < 25, n (%)	432 (30.1)
	≥ 25- < 30, n (%)	562 (39.2)
	≥ 30- < 40, n (%)	382 (26.7)
	≥ 40, n (%)	35 (2.4)
*Smoking status*	Current, n (%)	436 (30.4)
	Former, n (%)	824 (57.5)
	Never, n (%)	174 (12.1)
*Medication class in primary inhaler*	LABA, n (%)	112 (7.8)
	LAMA, n (%)	385 (26.8)
	LABA/LAMA, n (%)	357 (24.9)
	LABA/LAMA/ICS, n (%)	63 (4.4)
	ICS, n (%)	9 (0.6)
	ICS/LABA, n (%)	506 (35.3)
	Short-acting, n (%)	2 (0.1)
GOLD stage^a^	n (% non-missing)	801 (55.9)
	I, n (%)	189 (23.6)
	II, n (%)	440 (54.9)
	III, n (%)	139 (17.4)
	IV, n (%)	33 (4.1)
**Outcome measures**		
Clinical COPD Questionnaire (CCQ)	Mean (SD)	1.7 (1.1)
COPD Assessment Test (CAT)	Mean (SD)	13.6 (7.8)
Number of moderate exacerbations^b^	0, n (%)	1,113 (77.6)
	1, n (%)	167 (11.6)
	2, n (%)	72 (5.0)
	3, n (%)	37 (2.6)
	≥ 4, n(%)	45 (3.1)
Number of severe exacerbations^b^	0, n (%)	1,386 (96.7)
	1, n (%)	38 (2.6)
	2, n (%)	5 (0.3)
	3, n (%)	2 (0.1)
	≥ 4, n(%)	3 (0.2)

^a^GOLD classification of severity of airflow limitation was available for 801 participants

^b^32% of the exacerbation history information was retrieved from medical records, the remaining 68% was patient-reported

### Description of inhalation technique errors

**Fig. 1:**
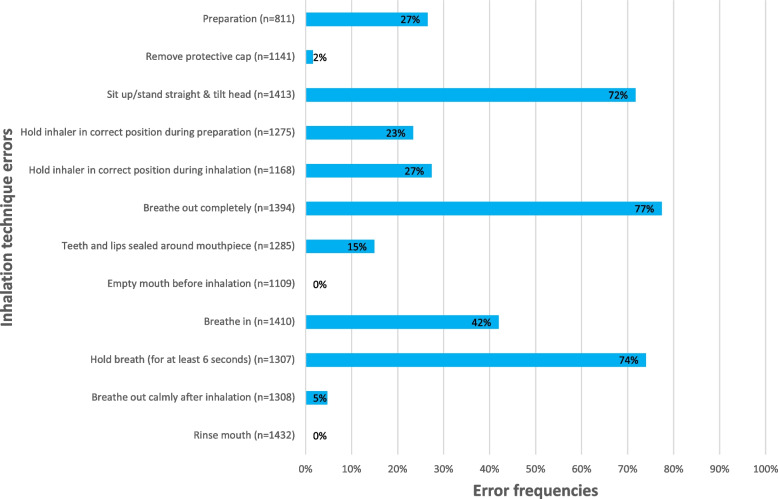
Error frequencies observed in the PIFotal study[8], grouped for all DPIs. Note: ‘Empty mouth before inhalation’ & ‘Rinse mouth’ were not reported so were excluded in the models

The most frequent errors made by over 70% of patients were ‘Sit up/stand straight & tilt head’ ‘Breathe out completely before inhalation’ ‘Hold breath (for at least 6 s)’ (Fig. 1).

### Associations of individual inhalation errors with health status and exacerbations

**Fig. 2:**
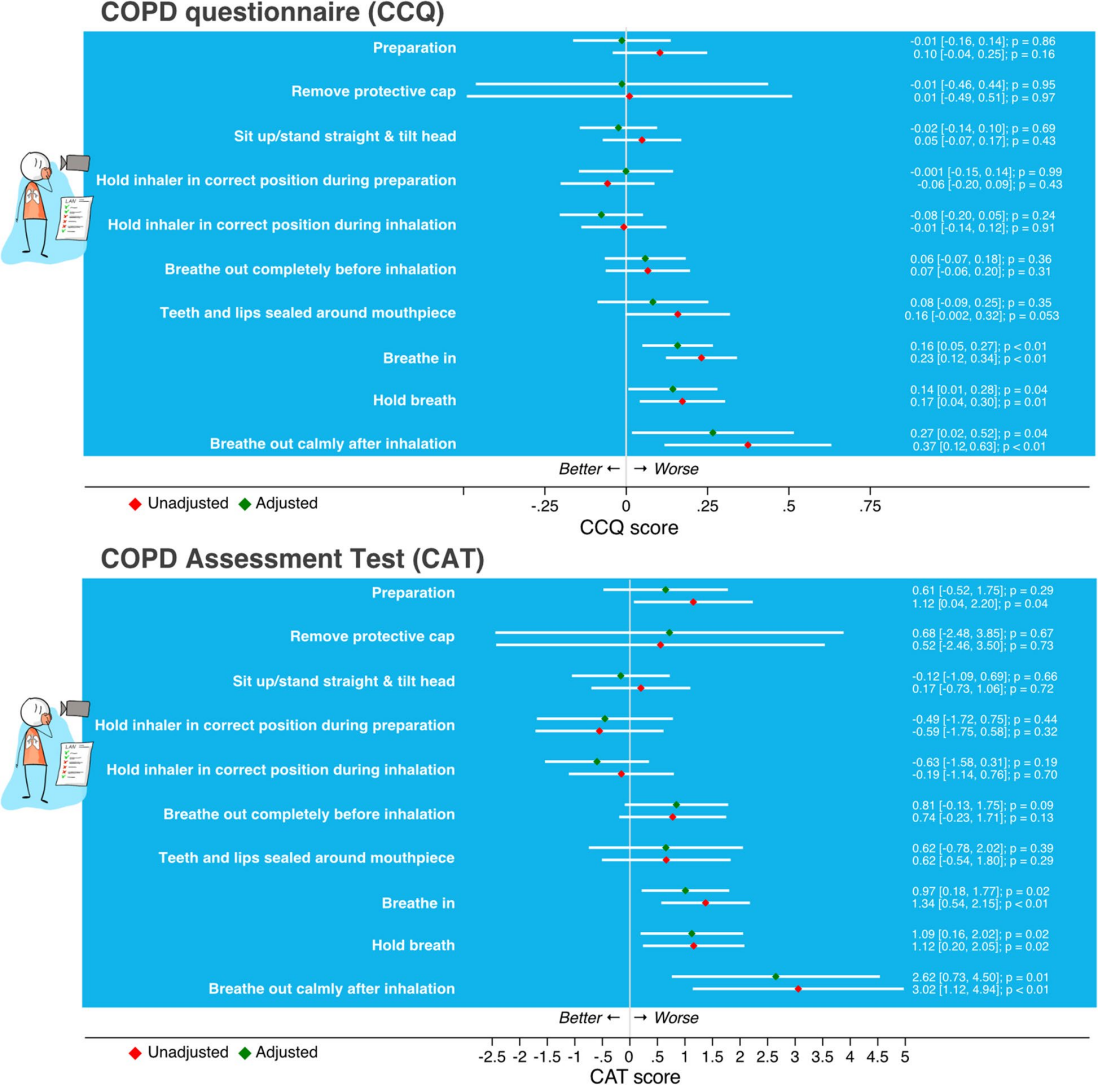
Associations between individual inhalation errors and the Clinical COPD Questionnaire (CCQ) and the COPD Assessment Test (CAT). For continuous outcomes (CCQ / CAT) linear multilevel models were used, reporting the estimate of the difference (β) in the absolute score (and 95% CI) between the dichotomous predictors (patients with and without the inhalation technique error)

**Fig. 3:**
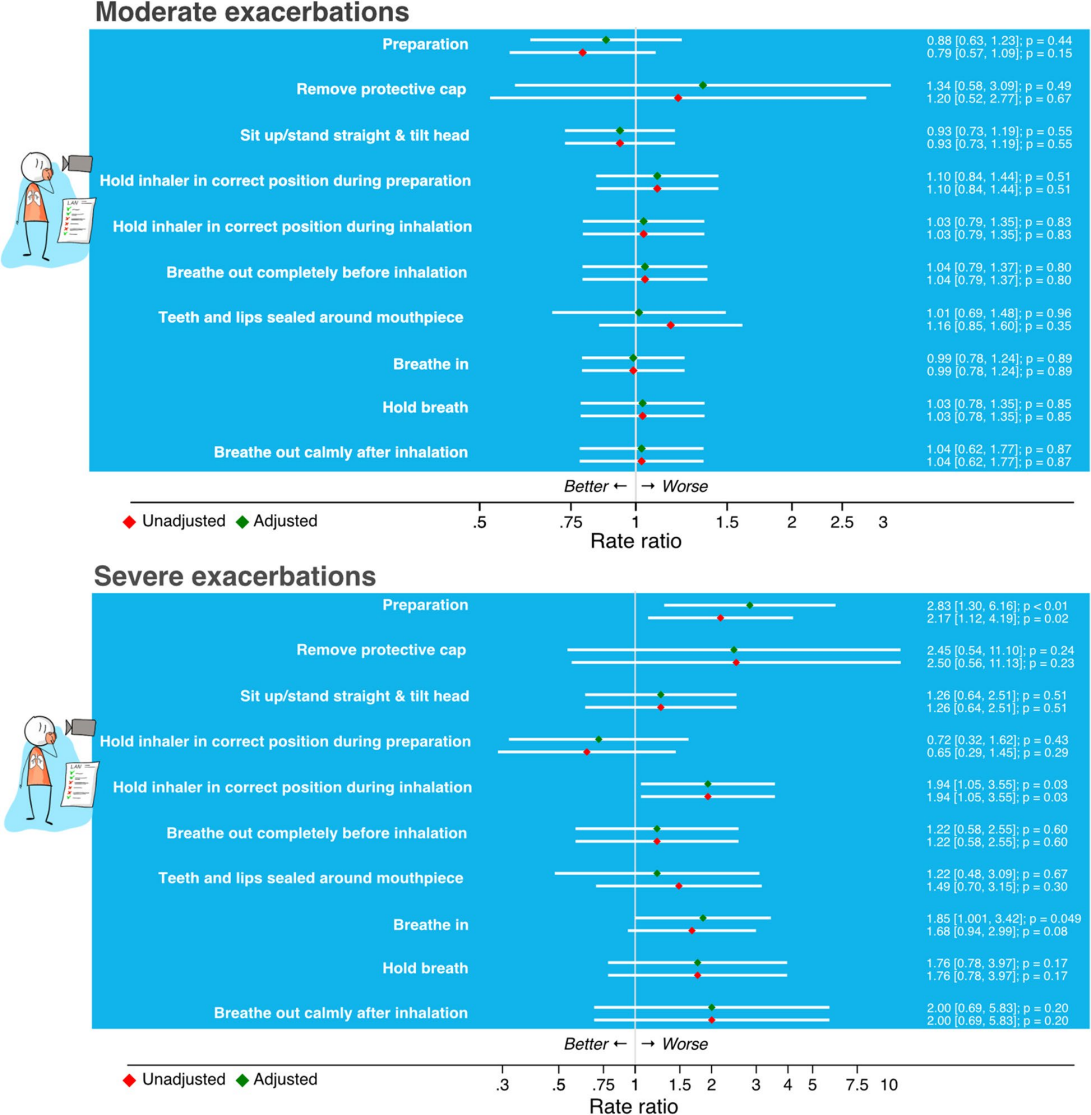
Associations between individual inhalation errors and the number of moderate or severe exacerbations in the last 12 months. For the number of exacerbations multilevel negative binomial regression models were used, reporting Rate Ratios (and 95% CI) between the dichotomous predictors (patients with and without the inhalation technique error)

In the adjusted analysis, both the CCQ and CAT score outcomes were significantly associated with the inhalation errors ‘Breathe in’ (CCQ β 0.16 95% CI [0.05, 0.27]); (CAT β 0.97 95% CI [0.18, 1.77]), ‘Hold breath’ (CCQ β 0.14 95% CI [0.01, 0.28]); CAT β 1.01 95% CI [0.16, 2.02]) and ‘Breathe out calmly after inhalation’ (CCQ β 0.27 95% CI [0.02, 0.52]); CAT β 2.62 95% CI [0.73, 4.50]) (Fig. 2). None of the inhalation errors were associated with the frequency of moderate exacerbations (Fig. 3). Patients with the errors ‘Preparation’ (RR = 2.83 95% CI [1.30, 6.16]), ‘Hold inhaler in correct position during inhalation’ (RR = 1.94 95% CI [1.05, 3.55]) or ‘Breathe in’ (RR = 1.85 95% CI [> 1.00, 3.42]) had on average significantly more severe exacerbations than patients without these errors (Fig. 3).

### Associations of error patterns with health status and exacerbations

As a part of our explorative analysis, we looked at the combinations of individual errors, i.e., error patterns, after removing the individual inhalation errors that were not significantly associated with any of the outcome measures. Consequently, four errors, ‘remove protective cap’, ‘sit up straight head tilted’, ‘position inhaler preparation’, and ‘sealed mouthpiece’ were of low importance (*p* > 0.2 for all four outcomes) in the individual error models and, therefore, not considered in the further analyses. The remaining six errors consisted of ‘preparation’, ‘hold inhaler in correct position during inhalation’, ‘breathe out completely before inhalation’, ‘breathe in’, ‘hold breath’, and ‘breathe out calmly after inhalation’.

Considering these six errors, many patterns were positively associated with the CCQ, meaning that patients with such patterns had on average a higher CCQ (i.e., worse health status) compared with patients with none of the six considered errors. Consistently, the error ‘Breathe in’ was prominent amongst the errors with the highest effect sizes, especially when combined with either error ‘breathe out completely before inhalation’ or ‘hold breath’ (Figure S1).

Within the patterns of six considered errors, the pattern combination of ‘breathing out completely before inhalation’, ‘breathing in’, and ‘hold breath’ was made by 15.2% of the patients. Patients with this pattern had on average a 0.20 higher CCQ score than patients with none of the six considered errors (95% CI [-0.06, 0.47], *p* = 0.13; Figure S1). The same trend was observed for the CAT score; patients with this pattern had on average a 1.48 higher CAT score than those patients without the six considered errors (95% CI [-0.47, 3.34], *p* = 0.14; Figure S1).

There were no patterns associated with the number of moderate exacerbations in the past 12 months (Figure S2). Concerning the number of severe exacerbations, patients with a pattern of ‘preparation’, ‘breathing out completely before inhalation’, ‘breathing in’, ‘holding breath’ (6.4% of the patients) had significantly more severe exacerbations compared to those without the six considered errors (RR = 5.70, 95% CI [1.00, 32.24]; *p* = 0.05; Figure S2).

**Fig. 4:**
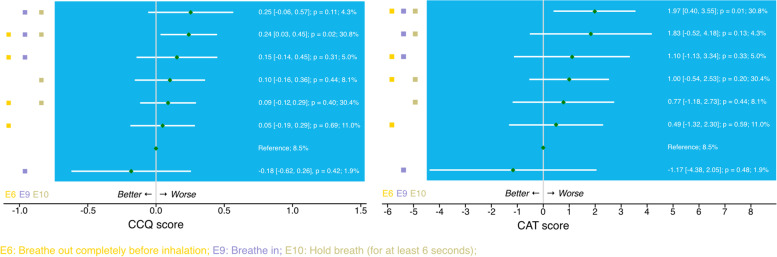
Model results of three-error patterns (‘breathe out completely before inhalation’, breathe in’, ‘hold breath (for at least 6 s)’) on Clinical COPD Questionnaire CCQ (*left*) and COPD Assessment Test CAT (*right*). For continuous outcomes (CCQ / CAT) linear multilevel models were used, reporting the estimate of the difference (β) in the absolute score (and 95% CI) between the categorical predictors (three-error pattern, with the reference group: patients with none of the three considered errors)

Subsequently, the analysis was repeated with common errors with the highest effect sizes, i.e., the three-error pattern of ‘breathing out completely before inhalation’, ‘breathing in’, ‘holding breath’, observed in 30.8% of the patients). Compared with patients with none of the three considered errors, patients with a pattern of errors ‘breathing out completely before inhalation’, breathing in’, holding breath’ had on average a 0.24 higher CCQ score (95% CI [0.03, 0.45], *p* = 0.02) and a 1.97 higher CAT score (95% CI [0.40, 3.55], *p* = 0.14; Fig. 4). In addition, patients with this pattern had on average more severe exacerbations compared with patients without these errors, with a RR of 2.78 (95% CI [0.62, 12.46], *p* = 0.18; Figure S3). None of the patterns were associated with the number of moderate exacerbations in the last 12-months.

### Associations between number of inhalation errors and health outcomes

**Fig. 5:**
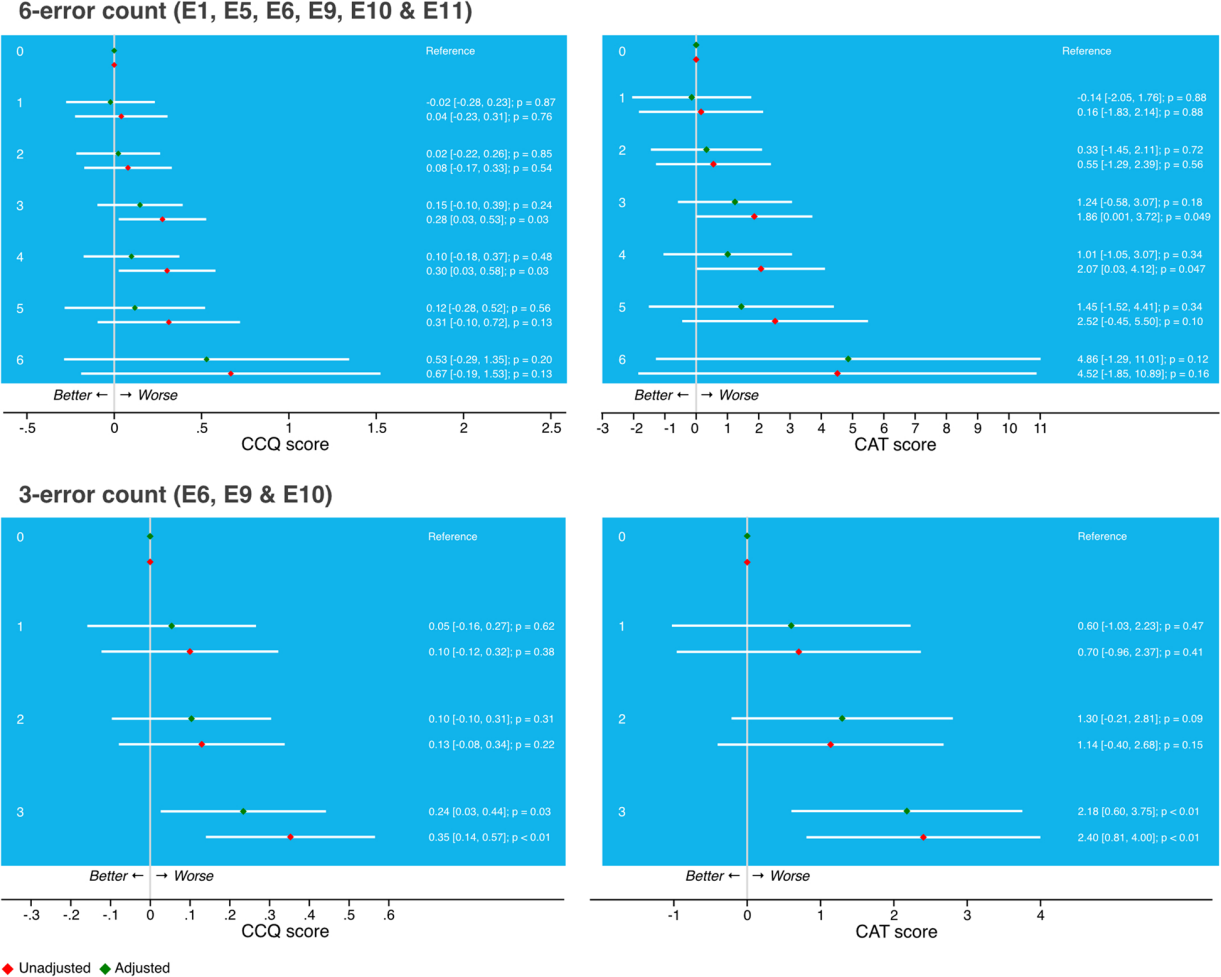
Model results of the number of inhalation errors (within the 6-error pattern top row, and within the 3-error pattern lower row) on Clinical COPD Questionnaire CCQ (left) and COPD Assessment Test CAT (right). For continuous outcomes (CCQ / CAT) linear multilevel models were used, reporting the estimate of the difference (β) in the absolute score (and 95% CI) between the categorical predictors (error count, with the reference group: patients with none of the considered errors)

A trend could be observed with CCQ or CAT worsening with increasing inhalation technique error count (Fig. 5). A significant association between the three-error count and health status was found. Compared with patients who did not show any of the three considered errors (i.e., no error in ‘breathe out completely before inhalation’ & ‘breathe in’ & ‘hold breath’), patients making these 3 errors had on average a 0.24 higher CCQ score (95% CI [0.06, 0.44]; *p* = 0.03) and a 2.18 higher CAT score (95% CI [0.60, 3.75]; *p* = 0.01; Fig. 5).

There was no association between the error count (within the six- and three considered errors) and the number of moderate exacerbations. Within the six-error count, patients making 5 errors had a significantly higher severe exacerbation rate compared to patients with none of the errors (RR = 7.28, 95% CI [1.07, 49.53]; *p* = 0.04; Figure S4).

The results from the sensitivity analysis, allowing more patterns by reducing the minimum frequency to 15 or 10 patients did not relevantly change the results.

Sensitivity analysis showed that the errors ‘breathe in’ and ‘breathe out calmly after inhalation’ revealed different associations for CCQ and CAT when looking into the effects in subgroups categorised based on the internal device resistances. Patients with the lowest resistance devices (low / medium low) showed higher CCQ- and CAT-scores when they made these errors compared with patients using DPIs with medium to high resistances (Figure S5).

Device resistance did not reveal significant differences in the associations with the error patterns/number of errors with the COPD outcome measures.

## Discussion

### Principle findings

**Fig. 6:**
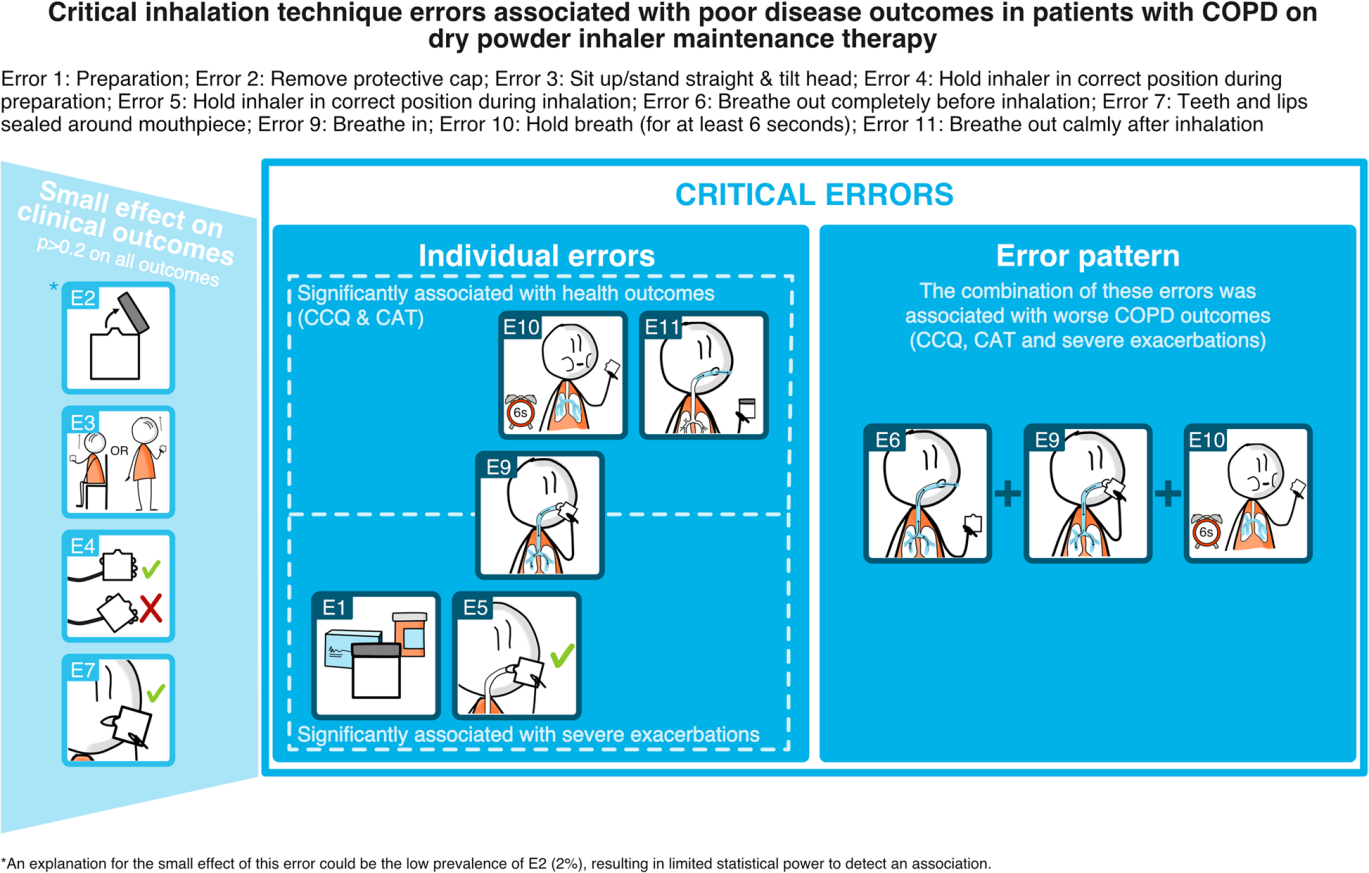
Critical inhalation technique errors associated with poor outcomes in patients with COPD on Dry Powder Inhalation maintenance therapy

This study assessed critical inhalation technique errors based on their association with health status and exacerbations using a comprehensive evaluation of the inhalation technique of 1,434 patients. Specifically, the inhalation technique errors of ‘Breathe in’, ‘Hold breath’ and ‘Breathe out calmly after inhalation’ were considered ‘critical errors’ based on their individual association with poorer health status. The errors ‘Preparation’, ‘Hold inhaler in correct position during inhalation’, and ‘Breathe in’ were deemed ‘critical’ considering their association with the number of severe exacerbations. Combinations of errors, especially ‘breathe in’ combined with ‘breathe out completely before inhalation’ and ‘hold breath’ (i.e., related to the inhalation manoeuvre) were associated with poorer health outcomes. Consistent with this finding, a trend could be observed with health status and severe exacerbations worsening with increasing error count. A graphical overview of the findings of this study can be found in Fig. 6.

### Interpretations and comparison with previous studies

The PIFotal study focused on the associations between DPI technique errors and clinical outcomes in COPD patients. This empirical approach is unique as most of the currently available literature identified errors as critical solely based on theory [5–7, 15, 16]. There is, however, a large cross-sectional study of asthma patients which looked at the association of the inhalation technique and disease control and exacerbation rate [13]. They found that insufficient inspiratory effort was common and associated with poorer health status, which is in line with our results showing ‘breathe in’ among the errors with the strongest association with poorer health status. This may be explained by the fact that the inspiratory effort, and corresponding PIF, influences the efficiency of the inhaler in lifting particles of the drug formulation from the drug chamber or capsule [22] Also, it is possible that drug particles are not being separated from their lactose carrier when the inspiratory flow is too weak [23] We, therefore, postulate that the ‘breathe in’ error and its association with poorer health status is plausible considering mechanisms of medication dose delivery into the lungs from DPIs.

In addition to the individual errors, the specific combinations of errors with an adverse impact on COPD outcomes revealed that HCPs should be aware of the interplay between the distinct steps of the inhalation manoeuvre and that comprehensive screening of the patients’ handling of the device is fundamental for effective therapy. Especially the error ‘breathe in’ in combination with ‘breathe out before inhalation’ or ‘holding breath (for at least 6 s)’ revealed larger associations than observed with the individual ‘breathe in’ error. Distinct inhalation technique steps may interfere and jointly contribute to adverse outcomes. The latter relevant pattern is in line with the general idea that breathing out before the inhalation, and holding breath (as long as possible) after inhalation are (in combination with sufficient inspiratory flow) essential for optimal drug delivery to the airways and enabling particles to be deposited in the peripheral areas by sedimentation [24, 25].

The observed error frequency in our sample tended to be higher compared to other studies. For example, the ‘Sit up/stand straight & tilt head’; ‘Breathe out completely before inhalation’; and ‘Hold breath’ occurred in 70.7%, 75.2%, and 67.4%, respectively. Whereas in CRITIKAL, the frequency of such generic errors was lower (34.3—34.6%, 26.2—32.4%, and 22.1—24.7%, for respectively Turbuhaler-Symbicort and Diskus-Seretide clusters) [13] Several other studies also report lower error frequencies compared to our study, e.g. the errors ‘Hold breath’ occurred in a range from 25–43.2%. ‘Breathe out before inhalation’ ranged from 33.3–54.7% [15, 26, 27]. These discrepancies may be explained by differences in demographic and clinical characterstics (such as age, co-morbidities and the inflammatory process [28]) between COPD and Asthma. A previous study revealed evidence suggesting that the orientation of inhalers (upright or leaning forward) contributes to higher oropharyngeal deposition in pMDIs but not in DPIs [29]. This finding potentially elucidates why the CRITICIKAL study classified head tilting as a ‘critical’ error among asthma patients using a DPI, whereas this error was not considered critical in this study involving patients with COPD using a DPI. Further, our extensive inhalation technique assessment may have resulted in higher error frequencies. While most other studies scored the errors during the visit, videos of the inhalation technique were recorded in this study and scored after the event. The recording was assessed and evaluated by two independent researchers, and a consensus meeting to reach an agreement was held when needed. This method allowed us to pause and replay the video, which increased focus on inhalation technique details which are difficult to assess during live observations (considering that a typical inhalation takes place in less than ten seconds). For daily clinical practice, where video assessments may not be possible, observation training for healthcare professsionals would be valuable particularly to improve the ability to identify the critical errors associated with health status. In addition, we used checklists from the Netherlands Lung Alliance to quantify the errors for all DPI’s and grouped them into distinct categories. This is in contrast to available literature where checklists from other studies were frequently used, or they used the instructions provided with the inhaler device [6] Finally, most of the studies only included a selection of the available DPIs, whereas the PIFotal study included data from 15 different DPIs and represented all medication classes.

Besides the error ‘Breathe in’, other critical errors were ‘Preparation’, ‘Hold inhaler in correct position during inhalation’, ‘Hold breath’ and ‘Breathe out calmly after inhalation’. Failure to perform the ‘Preparation’ step correctly may lead to no, or limited, availability of the inhaled drug. For single-dose inhalers, it is important to pierce the capsule only once during preparation, as instructed. The capsule aperture size should be within an optimal range to meet both the satisfied aerosol performance and minimised powder retention [30, 31] However, we observed that people tended to pierce the capsule multiple times which might increase the aperture size. The error ‘Hold inhaler in correct position during inhalation’ may cause the medication to fall out of the device resulting in a decreased amount of inhaled drug particles entering the lung and therefore reduced effectiveness. Incorrect positioning of the inhaler was mainly observed in the Ellipta device where the air vents were facing downward when inhaling. Although the manual stated that attention must be given to the position, with most patients being right-handed, the cap is shifted with the right hand and the device is taken to the mouth with the air vents down.

Interpretation of these findings concerning incorrect positioning of the inhaler must be done with caution, as we cannot fully rule-out bias by indication. More patients with the Ellipta device were on triple therapy, and although we adjusted for disease severity, the group making this error were likely to have a higher number of severe exacerbations. ‘Holding breath’ increases the amount of drug deposition in the lungs which is crucial in achieving maximum therapeutic benefit [32]. After inhaling, the medication needs to stick to the airway wall, and therefore needs time without airflow to be able to do so. The duration of breath-holding can be discussed. Several guidelines state 10 s, or at least as long as comfortable. In this study, we have used the commonly used 6 s as the cut-off. The criticality of the error ‘breathe out calmly after inhalation’ can be explained by coughing or breathing out too forcefully which may lead to insufficient drug deposition and exhaling of the active medication. We observed no association between health status and the error ‘remove protective cap’, which is peculiar since not removing the protective cap results in no medication arriving in the lungs. An explanation for this finding is the low prevalence of the said error, which accounts for less than 2%, resulting in limited power to detect an association.

The interaction effect of device resistance on the associations with errors ‘breathe in’ and ‘breathe out calmly after inhalation’ on the CCQ- and CAT- scores might be explained by the fact that patients with worse disease status may be more likely to get a prescription for particular treatments which may be available in lower internal resistance inhalers. Overall, subgroups of device resistances did not meaningfully modify the associations of error patterns/number of inhalation errors, which highlights the generalisability of the findings.

### Strengths and limitations of the study

This large, real-world design study of COPD patients, conducted in multiple countries and including many DPI types for maintenance therapy, provides strong empirical evidence for the definition of ‘critical’ inhalation technique errors by their association with health outcomes. Over 80% of the patient population revealed at least one critical inhalation technique error when handling their DPI, further stressing the importance of monitoring and tackling inhalation technique errors in daily clinical practice.

A limitation of our analyses is that we cannot exclude residual confounding, although our analyses were adjusted for an extensive set of potential confounders based on literature and clinical expertise.

Significant associations were identified in the cross-sectional study, between specific inhalation technique errors and health outcomes. Whilst not being able to conclude causality, or exclude the impact of unmeasured confounders, the analyses were adjusted for a comprehensive set of potential confounders based on the literature and clinical expertise. Such adjustments did not appear to have an impact on the statistical significance of the association between the errors and the outcomes, increasing confidence in the findings (Table S2).

**Fig. 7:**
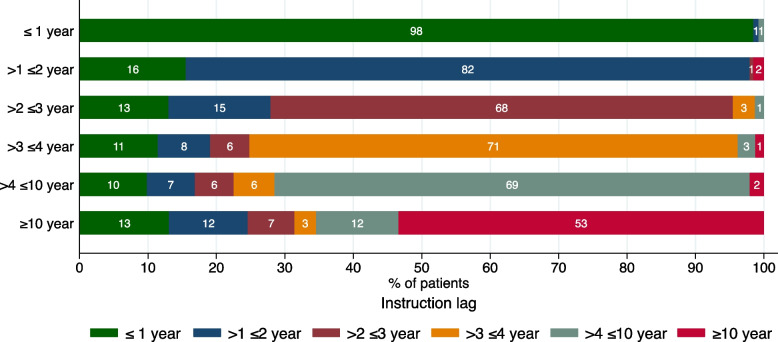
The relationship between the duration of inhaler use (y-axis) and the years since the last inhalation instruction

Although it is not a limitation, it is worth emphasising that this study was performed during the COVID-19 pandemic. This may have influenced some of the outcomes, for instance, the rate of exacerbations may not be entirely representative for a regular non-pandemic year [33], and may also present a bias in terms of patients willing to partake in research at this time. In addition, the pandemic may have delayed the regular check-ups and monitoring of inhalation technique thereby potentially influencing the proportion of observed errors. On the other hand, more than half of the patients who used their current inhaler for more than 2 years only received inhalation instructions when they started using the device (Fig. 7). Of note, 53% of the patients who used their current inhaler for more than 10 years had not been reinstructed in the last 10 years.

### Future recommendations

The findings from the PIFotal study provided evidence that clinical practice should focus on regular inhalation technique assessments, and indicate that there is a potential for patient-specific training targeting the critical errors. HCPs should be trained to improve the ability to identify ‘critical’ inhalation technique errors in patients with COPD using a DPI. Future prospective longitudinal studies should establish the efficacy of inhalation technique training targeting the critical errors and, more specifically, determine which inhalation steps show a strong retention effect post-training. In addition, it is of utmost importance that generic inhalation technique checklists are globally adopted to make the interpretation of results among studies more conclusive for HCPs. The study was conducted in patients with relatively stable COPD. To extrapolate results to a wider COPD population it would be interesting to complete this study when patients are experiencing an exacerbation.

## Conclusions

In a large COPD population, the errors ‘Breathe in’, ‘Hold breath (for at least 6 s)’ and ‘Breathe out calmly after inhalation’ were deemed critical based on their association with worse health status, whereas the errors ‘Preparation’, ‘Hold inhaler in correct position during inhalation’, and ‘Breathe in’ were deemed critical based on the association with severe exacerbations. Over 80% of the patients made one or more of the identified critical errors. Investigating combinations of errors associated with poorer COPD outcomes revealed that the various inhalation steps relating to the inhalation maneouvre in its entirety may be more clinically relevant. That said, COPD outcomes worsened with increasing error count. To improve COPD outcomes, HCPs should focus on all inhalation steps to eliminate as many (critical) errors as possible.

### Supplementary Information

**Additional file 1:** **TableS1.** Overview of DPIs included in the PIFotal study, the corresponding checklists for the error ‘breathe in’, by internal device resistance. **Table S2.** Overview of confounder candidates. **Table S3.** Overview of confounders included in the models. **Figure S1.** Model results of categorical error patterns on Clinical COPD Questionnaire (CCQ; *left*) and COPD Assessment Test (CAT; *right*). **Figure S2.** Model results of categorical error patterns on the number of moderate (*left*) and severe (*right*) exacerbations. **Figure S3.** Model results of three-error pattern combinations (‘breathe out before inhalation’, breathe in’, ‘hold breath (forat least 6 seconds)’) on moderate (*left*) and severe (*right*) exacerbations. **Figure S4.**Model results of the 6 error count on moderate (*left*) and severe exacerbations (*right*). **Figure S5.** Effect sizes of individual inhalation technique errors on the CCQ (*left*) and CAT (*right*), for different device resistances (low/medium-low, medium, medium-high/high).

## Data Availability

Data are available on reasonable request from the corresponding author JK.

## Abbreviations

CAT: COPD Assessment Test
CCQ: Clinical COPD Questionnaire
COPD: Chronic Obstructive Pulmonary Disease
DPI: Dry Powder Inhaler
GOLD: Global Initiative for Chronic Obstructive Lung Disease
HCP: Healthcare Professional
LAN: Lung Alliance Netherlands
PIF: Peak Inspiratory Flow
RR: Rate Ratio
