# Supplementary material for: Identifying critical inhalation technique errors in Dry Powder Inhaler use in patients with COPD based on the association with health status and exacerbations: findings from the multi-country cross-sectional observational PIFotal study
Source: BMC Pulm Med. 2023 Aug 17;23:302. doi: 10.1186/s12890-023-02566-6 (PMC10433653; doi:10.1186/s12890-023-02566-6)
Supplement: Supplementary file 1 — Additional file 1: TableS1. Overview of DPIs included in the PIFotal study, the corresponding checklists for the error ‘breathe in’, by internal device resistance. Table S2. Overview of confounder candidates. Table S3. Overview of confounders included in the models. Figure S1. Model results of categorical error patterns on Clinical COPD Questionnaire (CCQ; left) and COPD Assessment Test (CAT; right). Figure S2. Model results of categorical error patterns on the number of moderate (left) and severe (right) exacerbations. Figure S3. Model results of three-error pattern combinations (‘breathe out before inhalation’, breathe in’, ‘hold breath (forat least 6 seconds)’) on moderate (left) and severe (right) exacerbations. Figure S4.Model results of the 6 error count on moderate (left) and severe exacerbations (right). Figure S5. Effect sizes of individual inhalation technique errors on the CCQ (left) and CAT (right), for different device resistances (low/medium-low, medium, medium-high/high). [file 12890_2023_2566_MOESM1_ESM.docx]

# Supplementary material

**Table S1**. Overview of DPIs included in the PIFotal study, the corresponding checklists for the error ‘breathe in’, by internal device resistance.

| **Inhaler** | **Resistance** | **Description ‘breathe in’ step**  **translated from Netherlands Lung Alliance (LAN) or the Aerosol Drug Management Improvement Team** |
| --- | --- | --- |
| Breezhaler | Low | Inhale calmly, completely with steady moderate force. The inhalation is strong enough when the capsule vibrates during the inhalation. |
| Cyclohaler (Aerolizer) | Low | Inhale calmly, completely with steady moderate force. The inhalation is strong enough when the capsule vibrates during the inhalation. |
| Ellipta | Medium-low | Inhale as deeply as possible with steady, moderate force. |
| Diskus | Medium-low | Inhale as deeply as possible with steady, moderate force. |
| Elpenhaler | Medium-low | Inhale as deeply as possible with steady, moderate force. |
| Turbuhaler – Symbicort | Medium | Breathe in forcefully and deeply. |
| Genuair | Medium | Inhale deeply with powerful force. Within the first second, you will hear a ‘click’. Do not stop inhaling. |
| Novolizer | Medium | Inhale deeply with powerful force. Within the first second, you will hear a ‘click’. Do not stop inhaling. |
| Spiromax | Medium | Inhale as deeply as possible with powerful force. |
| Forspiro | Medium | Inhale as deeply as possible with steady, moderate force. |
| Clickhaler | Medium | Breathe in as briskly, quickly, and deeply as you can until your lungs are full. |
| Nexthaler | Medium-high | Inhale deeply with powerful force. Within the first second, you will hear a ‘click’. Do not stop inhaling. |
| Easyhaler | Medium-high | Inhale as deeply as possible with steady, moderate force. |
| Turbuhaler – Oxis/Pulmicort/Budesonide | Medium-high | Breathe in forcefully and deeply. |
| Zonda | High | Inhale deeply with powerful force. The inhalation is strong enough when you can hear the capsule making a vibrating noise. |
| Handihaler | High | Inhale deeply with powerful force. The inhalation is strong enough when the capsule vibrates (rattles) during the inhalation. |

**Table S2**. Overview of confounder candidates

| **All models:** |  |  |
| --- | --- | --- |
| **Country of residence** | Australia, n (%)  Spain, n (%)  Greece, n (5)  The Netherlands, n (%)  Poland, n (%)  Portugal, n (%) | 19 (1.3)  432 (30.1)  196 (13.7)  468 (32.6)  113 (7.9)  206 (14.4) |
| **Age** | Mean (SD) | 69.2 (9.3) |
| **Sex** | Female, n (%)  Male, n (%) | 718 (50.1)  716 (49.9) |
| **Body Mass Index** | Mean (SD) | 27.8 (5.3) |
| **Smoking status** | Current, n (%)  Former, n (%)  Never, n (%) | 436 (30.4)  824 (57.5)  174 (12.1) |
| **Educational level** | Primary, n (%)  Secondary, n (%)  Post-secondary vocational, n (%)  University, n (%) | 436 (30.4)  301 (21.0)  368 (25.7)  327 (22.8) |
| **Medication class in device** | LABA, n (%)  LAMA, n (%)  LABA/LAMA, n (%)  LABA/LAMA/ICS, n (%)  ICS, n (%)  ICS/LABA, n (%)  Short-acting, n (%) | 112 (7.8)  385 (26.8)  357 (24.9)  63 (4.4)  9 (0.6)  506 (35.3)  2 (0.1) |
| **Overall COPD regimen** | Triple Therapy, n (%)  ICS + (LAMA or LABA), n (%)  LAMA+LABA, n (%)  LAMA or LABA or ICS mono, n (%) | 331 (23.1)  419 (29.2)  359 (25.0)  325 (22.7) |
| **SARS-COV-2 history** | Negative, n (%)  Positive - Managed at home, n (%)  Positive - Managed at hospital, n (%) | 1353 (95.1)  56 (3.9)  13 (0.9) |
| **Lung comorbidity** | n (%) | 122 (8.5) |
| **Cardiovascular comorbidity** | n (%) | 642 (45.0) |
| **Depression** | n (%) | 288 (20.2) |
| **Anxiety** | n (%) | 344 (24.1) |
| **Diabetes mellitus** | n (%) | 295 (20.7) |

**Table S3**. Overview of confounders included in the models

| Outcome | Predictor | Identified confounders |
| --- | --- | --- |
| CCQ | Preparation | Anxiety; BMI; Cardiovascular comorbidity; Country of residence; Depression; Lung comorbidity; Medication class; Smoking status; Medication regimen; |
|  | Remove protective cap | Anxiety; BMI; Cardiovascular comorbidity; Country of residence; Depression; Diabetes; Educational level; Sex; Lung comorbidity; Medication class; Device resistance; Smoking status; |
|  | Sit up/stand straight & tilt head | Anxiety; BMI; Country of residence; Educational level; Device resistance; Medication regimen; |
|  | Hold inhaler in correct position during preparation | Age; Anxiety; BMI; Cardiovascular comorbidity; Country of residence; Depression; Diabetes; Educational level; Sex; Lung comorbidity; Medication class; Device resistance; SARCOV2; Medication regimen; |
|  | Hold inhaler in correct position during inhalation | Country of residence; Depression; Device resistance; Medication regimen; |
|  | Breathe out completely before inhalation | Anxiety; BMI; Depression; Diabetes; Educational level; Medication class; Medication regimen; |
|  | Teeth and lips sealed around mouthpiece | Anxiety; Country of residence; Diabetes; Sex; Medication class; |
|  | Breathe in | Anxiety; Country of residence; |
|  | Hold breath | Country of residence; Depression; |
|  | Breathe out calmly after inhalation | Anxiety; Country of residence; |
|  | Categories of 6-error patterns (imputed) | Anxiety; BMI; Cardiovascular comorbidity; Country of residence; Depression; Diabetes; Educational level; Sex; Lung comorbidity; Medication class; Device resistance; SARCOV2; Smoking status; Medication regimen; |
|  | Categories of 3-error patterns (imputed) | Anxiety; BMI; Country of residence; Depression; Diabetes; Sex; Lung comorbidity; Medication class; Medication regimen; |
| CAT | Preparation | Depression; Device resistance; |
|  | Remove protective cap | Anxiety; BMI; Cardiovascular comorbidity; Country of residence; Depression; Medication class; Device resistance; |
|  | Sit up/stand straight & tilt head | Anxiety; BMI; Country of residence; Educational level; Device resistance; Smoking status; Medication regimen; |
|  | Hold inhaler in correct position during preparation | Anxiety; BMI; Cardiovascular comorbidity; Educational level; Medication class; |
|  | Hold inhaler in correct position during inhalation | Country of residence; Depression; Device resistance; Medication regimen; |
|  | Breathe out completely before inhalation | BMI; Depression; Medication regimen; |
|  | Teeth and lips sealed around mouthpiece | Depression; Medication class; Device resistance; Smoking status; Medication regimen; |
|  | Breathe in | Anxiety; Smoking status; |
|  | Hold breath | Country of residence; Depression; |
|  | Breathe out calmly after inhalation | Anxiety; |
|  | Categories of 6-error patterns (imputed) | Age; Anxiety; BMI; Cardiovascular comorbidity; Country of residence; Depression; Diabetes; Educational level; Lung comorbidity; Medication class; Device resistance; SARCOV2; Smoking status; Medication regimen; |
|  | Categories of 3-error patterns (imputed) | Anxiety; BMI; Country of residence; Depression; Medication class; SARCOV2; Medication regimen; |
| Moderate exacerbations | Preparation | Medication regimen; |
|  | Remove protective cap | Country of residence; |
|  | Sit up/stand straight & tilt head | - |
|  | Hold inhaler in correct position during preparation | - |
|  | Hold inhaler in correct position during inhalation | - |
|  | Breathe out completely before inhalation | - |
|  | Teeth and lips sealed around mouthpiece | Device resistance; |
|  | Breathe in | - |
|  | Hold breath | - |
|  | Breathe out calmly after inhalation | - |
|  | Categories of 6-error patterns (imputed) | Cardiovascular comorbidity; Country of residence; Medication regimen; |
|  | Categories of 3-error patterns (imputed) | Country of residence; Medication regimen; |
| Severe exacerbations | Preparation | Device resistance; |
|  | Remove protective cap | Cardiovascular comorbidity; Educational level; |
|  | Sit up/stand straight & tilt head | - |
|  | Hold inhaler in correct position during preparation | Lung comorbidity; |
|  | Hold inhaler in correct position during inhalation | - |
|  | Breathe out completely before inhalation | - |
|  | Teeth and lips sealed around mouthpiece | Device resistance; |
|  | Breathe in | Device resistance; |
|  | Hold breath | - |
|  | Breathe out calmly after inhalation | - |
|  | Categories of 6-error patterns (imputed) | Age; Anxiety; BMI; Cardiovascular comorbidity; Country of residence; Diabetes; Educational level; Sex; Lung comorbidity; Medication class; Device resistance; SARCOV2; Smoking status; Medication regimen; |
|  | Categories of 3-error patterns (imputed) | Cardiovascular comorbidity; Educational level; Sex; Lung comorbidity; Medication class; Device resistance; |

**Figure S1.** Model results of categorical error patterns on Clinical COPD Questionnaire (CCQ; *left*) and COPD Assessment Test (CAT; *right*).

*For continuous outcomes (CCQ / CAT) linear multilevel models were used, reporting the estimate of the difference (β) in the absolute score (and 95% CI) between the categorical predictors (six-error pattern, with the reference group: patients with none of the six considered errors).*

**Figure S2.** Model results of categorical error patterns on the number of moderate (*left*) and severe (*right*) exacerbations.

*For the number of exacerbations multilevel negative binomial regression models were used, reporting Rate Ratios (and 95% CI) between the categorical predictors (six-error pattern, with the reference group: patients with none of the six considered errors).*

**Figure S3**. Model results of three-error pattern combinations (‘breathe out before inhalation’, breathe in’, ‘hold breath (for at least 6 seconds)’) on moderate (*left*) and severe (*right*) exacerbations.

*For the number of exacerbations multilevel negative binomial regression models were used, reporting Rate Ratios (and 95% CI) between the categorical predictors (three-error pattern, with the reference group: patients with none of the three considered errors).*

**Figure S4**. Model results of the 6 error count on moderate (*left*) and severe exacerbations (*right*).

*For the number of exacerbations multilevel negative binomial regression models were used, reporting Rate Ratios (and 95% CI) between the* *categorical predictors (error count, with the reference group: patients with none of the considered errors).*

**Figure S5**. Effect sizes of individual inhalation technique errors on the CCQ (*left*) and CAT (*right*), for different device resistances (low/medium-low, medium, medium-high/high)

*For continuous outcomes (CCQ / CAT) linear multilevel models were used, reporting the estimate of the difference (β) in the absolute score (and 95% CI) between the dichotomous predictors (patients with and without the inhalation technique error).*
